# Total *versus* partial posterior fundoplication in the surgical repair of para-oesophageal hernias: randomized clinical trial

**DOI:** 10.1093/bjsopen/zrac034

**Published:** 2022-05-02

**Authors:** Apostolos Analatos, Mats Lindblad, Christoph Ansorge, Lars Lundell, Anders Thorell, Bengt S. Håkanson

**Affiliations:** Division of Surgery, Department of Clinical Science, Intervention and Technology (CLINTEC), Karolinska Institutet, Stockholm, Sweden; Department of Surgery, Nyköping Hospital, Nyköping, Sweden; Centre for Clinical Research Sörmland, Uppsala University, Uppsala, Sweden; Division of Surgery, Department of Clinical Science, Intervention and Technology (CLINTEC), Karolinska Institutet, Stockholm, Sweden; Department of Upper Abdominal Surgery, Karolinska University Hospital, Stockholm, Sweden; Division of Surgery, Department of Clinical Science, Intervention and Technology (CLINTEC), Karolinska Institutet, Stockholm, Sweden; Department of Surgery, Nyköping Hospital, Nyköping, Sweden; Division of Surgery, Department of Clinical Science, Intervention and Technology (CLINTEC), Karolinska Institutet, Stockholm, Sweden; Department of Surgery, Odense University Hospital, Odense, Denmark; Karolinska Institutet, Department of Clinical Sciences, Danderyds Hospital and Department of Surgery and Anaesthesiology, Ersta Hospital Stockholm, Stockholm, Sweden; Karolinska Institutet, Department of Clinical Sciences, Danderyds Hospital and Department of Surgery and Anaesthesiology, Ersta Hospital Stockholm, Stockholm, Sweden

## Abstract

**Background:**

Fundoplication is an essential step in para-oesophageal hernia (POH) repair, but which type minimizes postoperative mechanical complications is controversial.

**Methods:**

This was a randomized, double-blind clinical trial conducted between May 2009 and October 2018. Patients with symptomatic POH were allocated to either a total (Nissen) or a posterior partial (Toupet) fundoplication after hernia reduction and crural repair. The primary outcome was dysphagia (Ogilvie dysphagia scores) at 6 months postoperatively. Secondary outcomes were peri- and postoperative complications, swallowing difficulties assessed by the Dakkak dysphagia score, gastro-oesophageal reflux, quality of life (QoL), and radiologically confirmed hernia recurrence.

**Results:**

A total of 70 patients were randomized to a Nissen (*n* = 32) or a Toupet (*n* = 38) fundoplication. Compared with baseline, Ogilvie dysphagia scores were stable at the 3- and 6-month follow-up in the Nissen group (*P* = 0.075 and 0.084 respectively) but significantly improved in the Toupet group (from baseline mean (s.d.): 1.4 (1.1) to 0.5 ( 0.8) at 3 months, and 0.5 (0.6) at 6 months; *P* = 0.003 and *P* = 0.001 respectively). At 6 months, Dakkak dysphagia scores were significantly higher in the Nissen group than in the Toupet group (mean (s.d.): 10.4 (7.9) *versus* 5.1 (7.2); *P* = 0.003). QoL scores improved throughout the follow-up. However, at 3 and 6 months postoperatively, the absolute median improvement (⍙) from preoperative values in the mental component scores of the Short Form-36 QoL questionnaire was significantly higher in the Toupet group (median (i.q.r.): 7.1 (−0.6 to 15.2) *versus* 1.0 (−5.4 to 3.3) at 3 months, and 11.2 (1.4 to 18.3) *versus* 0.4 (−9.4 to 7.5) at 6 months; (*P* = 0.010 and 0.003 respectively)). At 6 months, radiologically confirmed POH recurrence occurred in 11 of 24 patients (46 per cent) of the Nissen group and in 15 of 32 patients (47 per cent) of the Toupet group (*P* = 1.001).

**Conclusions:**

A partial posterior wrap (Toupet fundoplication) showed reduced obstructive complications and improved QoL compared with a total (Nissen) fundoplication following POH repair.

Registration number: NCT04436159 (http://www.clinicaltrials.gov)

## Teaser

In the present randomized, double-blind clinical trial in patients undergoing surgery for para-oesophageal hernia, the addition of a Nissen fundoplication to the hiatal reconstruction was associated with more mechanical side effects in the form of swallowing difficulties compared with a Toupet procedure.

## Introduction

Laparoscopic repair has been established as a safe and effective treatment for symptomatic para-oesophageal hernia (POH)^1–4^. The crucial steps of all surgical repairs include reducing the hernia and restoring normal anatomy, excision of the hernia sac, and a tension-free mobilization of the gastro-oesophageal junction (GOJ) in the abdominal cavity, followed by the closure of the oesophageal hiatus^5^. Based on available data^6,7^, the routine performance of fundoplication is generally recommended as a means of retaining the stomach below the diaphragm, as well as minimizing risks of reflux. In refining surgical strategies, the risk of complications and mechanical side effects should influence the type of fundoplication (partial or total) selected^8–10^. This might be particularly relevant for patients without gastro-oesophageal reflux disease (GORD), accounting for 30 per cent of patients undergoing surgery for POH^11–15^.

The diagnosis of a pre-existing GORD may be challenging in clinical practice, especially in fragile patients or in acute presentations requiring emergency surgery. Previous studies^16–18^ have shown a trade-off between dysphagia, other mechanical side effects, and control of reflux symptoms between Nissen and Toupet fundoplication in patients with chronic GORD. Lower rates of objectively documented acid reflux after Nissen fundoplication must be balanced against a lower incidence of dysphagia after Toupet fundoplication^16–20^. There are no definitive data, however, to address the effect of fundoplication after POH surgery. The present study aimed to test differences in obstructive complications comparing patients randomized to a total (Nissen) or a posterior partial (Toupet) fundoplication on a repositioned GOJ after laparoscopic POH repair.

## Methods

### Ethics

The study protocol was approved by the regional ethics committee (Stockholm) under approval number 2008/179-31. The study was carried out following the Helsinki Declaration and registered at ClinicalTrials.gov (identifier NCT04436159). After receiving oral and written information, patients who were willing to participate gave their written informed consent. The original study protocol and an English-language version are reported in Supplementary material. This report was written following the CONSORT guidelines^21^. The CONSORT checklist is available as Supplementary material.

### Eligible patients

Patients requiring emergency or elective surgery for symptomatic POH at Ersta Hospital or Karolinska University Hospital were eligible for inclusion. Only POH with 4 cm or larger in axial length was included. POH classification^22^ encompassed type II (pure para-oesophageal), type III (combined sliding and para-oesophageal), and type IV (in which organs other than the stomach entered the hernia sac). Exclusion criteria were axial sliding hiatal hernia (type I^22^), patients aged under 18 years, previous surgery for hiatal hernia, ASA score IV, or above, oesophageal achalasia, or other oesophageal motility disorders, Zollinger–Ellison syndrome, any malignant tumours, inability to give informed consent, and unwillingness to participate in the study.

### Pre- and postoperative investigations

A complete baseline data set was retrieved for patients scheduled for elective operations, unobtainable before emergency procedures. Otherwise, preoperative investigations included upper gastrointestinal (GI) endoscopy, high-resolution oesophageal manometry, ambulatory 24-h pH monitoring, radiology (Barium swallow radiography or CT), and quality of life (QoL) questionnaires (Short Form-36; SF-36).

Two validated scores were used to assess and record swallowing difficulties. The Ogilvie dysphagia score is a five-grade scale (0–4) defined as: 0, ability to eat ordinary diet; 1, ability to swallow some solid food; 2, ability to swallow semisolids; 3, ability to swallow liquids; and 4, total inability to swallow^23^. The Dakkak dysphagia score^24,25^ is a questionnaire for assessing benign dysphagia, including nine questions regarding the frequency (always, sometimes, or never) of swallowing difficulties with different food consistencies (liquid, semisolid, and solid foods). The final score ranges from 0 to 45, where 45 represents the most severe dysphagia.

QoL was assessed with the Swedish version of the validated SF-36^26,27^. Data are presented as physical and mental component scores (PCS and MCS respectively). Each subscale is scored on a 0 to 100 range so that higher scores reflect more favourable health status.

A high-resolution, solid-state system (ManoScan™, Medtronic, Synmed, Stockholm, Sweden) was used for standard oesophageal manometry. Oesophageal peristalsis was assessed in response to repeated water swallows, and the position and characteristics of the lower oesophageal sphincter (LOS) were analysed to exclude any specific oesophageal motor abnormalities and to localize the LOS.

Ambulatory 24-h pH monitoring was performed with a VersaFlex™ catheter system (single-use, one sensor with a diameter of 1.6 mm; Medtronic, Synmed, Stockholm, Sweden). The oesophageal pH probe was placed 5 cm above the upper border of the LOS as determined by manometry. The total time of oesophageal acid exposure (pH <4) was reported as a percentage of the total recorded time (Reflux Software™, Medtronic, Synmed, Stockholm, Sweden).

During follow-up, some investigations were repeated at 1 month (QoL and dysphagia scoring), 3 months (QoL and dysphagia scoring), and 6 months after the index surgery (QoL, dysphagia scoring, pH monitoring, and radiology).

### Randomization and blinding

Patients were stratified according to sex and BMI. A computer-generated randomization list in blocks of 10 was used. The randomization process was initiated after inducing general anaesthesia. Groups allocation was provided by opening a sealed envelope specifying the assigned group. The surgical report, including information on the type of POH repair and the fundoplication performed, was not included in the digital patient chart. Instead, a hard copy was printed and kept in a sealed envelope, filed in a locked archive to keep patients, staff, and clinical assessors blinded to the study group allocation. None of the participating surgeons carried out any of the postoperative analyses. Blinding was maintained during the follow-up.

### Surgical procedure

All involved surgeons were experienced in antireflux surgery (at least 25 procedures performed). Laparoscopy was the primary approach used throughout the trial, except for emergency procedures, where the surgeon’s experience guided the choice. All procedures included full mobilization and reduction of the hernia sac in the abdomen. The mediastinal oesophagus was dissected and mobilized to allow at least 3 cm of the lower oesophagus to remain below the hiatus in the abdominal cavity in a tension-free fashion. The gastric fundus was mobilized as necessary to perform the fundoplication. Vagal nerve branches were identified and kept within the gastric wrap. The explored hiatus was repaired by approximating the crura posterior to the oesophagus with at least three simple, non-absorbable, interrupted sutures with a stitch-to-stitch distance of 5–8 mm (2-0 GORE-TEX^®^ sutures). Anterior sutures were placed when necessary to obtain an adequate hiatal anatomy reconstruction.

When approximation of crurae was considered inadequate, a Crura-Soft^®^ large mesh was fitted onto the crus behind the oesophagus and secured with sutures and staples. No oesophageal bougie was placed during POH repair or fundoplication procedure. In patients undergoing a Nissen fundoplication, the right and left part of the gastric wrap was approximated in front of the oesophagus with three interrupted sutures attaining a length of the wrap of 2 cm at the most. At least one suture included the oesophageal muscle wall.

In patients allocated to a Toupet fundoplication, the gastric wrap was pulled around the posterior part of the distal oesophagus to encircle the GOJ by approximately 200 degrees. The gastric wrap was anchored dorsally to the left and right crus, both with three simple sutures. Finally, the wrap was sutured to the right and left sides of the anterior oesophageal wall respectively.

### Outcomes

The primary outcome was the incidence of swallowing difficulties assessed by the Ogilvie score at 6 months after surgery. Secondary outcomes were perioperative complications^28^ recorded out to 30 days after surgery and defined according to the Clavien–Dindo classification^28^, duration of hospital stay, postoperative swallowing difficulties assessed by the Dakkak dysphagia score, acid reflux control, QoL, and radiologically confirmed hiatal hernia recurrence (defined as any part of the stomach above the hiatal plane).

### Statistics and power calculation

Continuous variables are presented as median and interquartile range (i.q.r.) or means with standard deviation (s.d.). An intention-to-treat analysis was applied. Comparisons of parametric data were performed with a two-sided Student’s *t* test. A Mann–Whitney *U* test, Wilcoxon matched pairs test, or Friedman’s test was applied for non-parametric data. Categorical variables were reported as numbers with percentages and compared with the Chi-squared or Fisher’s exact test when appropriate. Additional analysis of absolute differences (⍙) between pre-and postoperative values was performed for the Ogilvie and SF-36 scores. A *P* value <0.05 was considered statistically significant. Statistical analysis was performed with the statistical software package SPSS^®^, version 26.0 (IBM, Armonk, New York, USA).

### Sample size

Based on previous randomized trials, a total fundoplication (Nissen)^29,30^ showed a 30 per cent incidence of postoperative dysphagia (defined as an Ogilvie score of 1 or higher) in the early postoperative interval. Conversely, less than 15 per cent of patients with chronic GORD complained of symptoms after having a Toupet repair^29,30^. Considering a 50 per cent decrease in Ogilvie scores at 6 months in the Toupet group compared with the Nissen group, a total of 50 patients were required to provide the study with at least 80 per cent power and given a 95 per cent c.i. By expecting high dropout rates (20 per cent) due to patient characteristics, 70 patients were finally estimated for inclusion.

## Results

### Patients

**Fig. 1:**
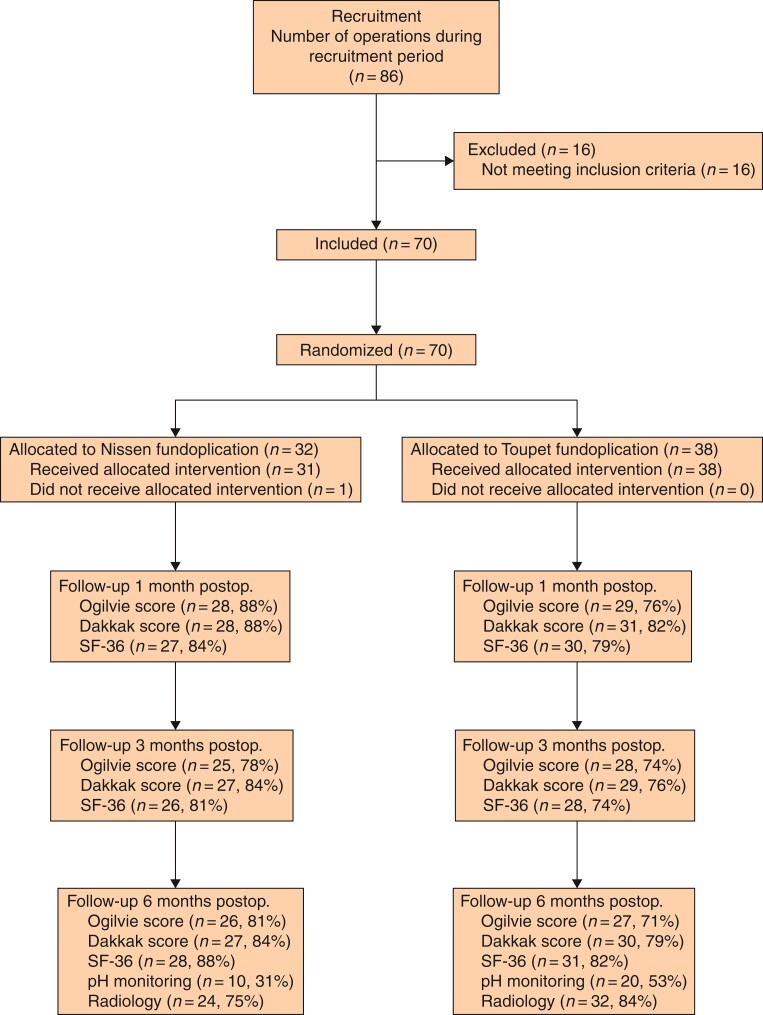
Flowchart of patients enrolled in the trial

The present trial was conducted from 4 May 2009 through 15 October 2018. A total of 86 consecutive patients undergoing POH repair were screened for eligibility; 70 patients were enrolled, and randomized to undergo a Nissen fundoplication (*n* = 32) or a Toupet fundoplication (*n* = 38). The CONSORT flowchart is shown in *Fig. 1*.

The number and percentages of patients completing the prescheduled follow-up investigations are given within brackets at various time points.

Groups were balanced for baseline characteristics, and both showed substantial co-morbidity rates (*Table 1*). A total of 39 of 70 patients (56 per cent) received proton pump inhibitors (PPIs) before the surgical procedure. Emergency surgery was performed in seven patients of the Nissen group and in six of the Toupet group (*P* = 0.782). Preoperative ambulatory 24-h pH monitoring was available in 31 patients (13 in the Nissen group and 18 in the Toupet group) and showed increased rates of oesophageal acid exposure in both groups (*Table 1*). Some elective patients did not get tested due to reluctance to undergo the investigation. Most POHs were classified as type III, and almost 1 of 5 of all procedures were performed in an emergency, or following an acute POH presentation. The median (i.q.r.) axial length of hernia was 7 cm (5–11 cm) in the Nissen group compared with 8 cm (6–11 cm) in the Toupet group (*P* = 0.373).

**Table 1:** Clinical and demographic preoperative characteristics in patients undergoing surgery for para-oesophageal hernia

	Nissen (*n* = 32)	Toupet (*n* = 38)	*P*
**Participating centre**			
Ersta Hospital/Karolinska Hospital	24/8	24/14	0.288*
**Sex**			
Male/female	10/22	10/28	0.649*
**Age (years), mean (s.d.)**	66 (9)	67 (10)	0.494†
**Co-morbidities**	Total *n* = 14	Total *n* = 10	0.126*
Cardiovascular	10	7	
Respiratory	1	1	
Diabetes	2	1	
Miscellaneous	1 (kidney disease)	1 (previous cardiac transplantation)	
**PPI intake (yes/no/missing)**	17/11/4	22/9/7	0.479*
**POH type**			0.286*
II	4	2	
III	21	23	
IV	7	13	
**Axial length of hernia (cm), median (i.q.r.)**	7 (5–11)	8 (6–11)	0.373†
**24-h total acid reflux (%)**	10.9 (4.3–16.3)	8.0 (0.2–11.4)	0.149†
**median (i.q.r.)**	(*n* = 13)	(*n* = 18)	
**Level of emergency**			0.782*
Elective	25	32	
Urgent	3	3	
Acute	4	3	

Patients were randomized to either a Nissen or Toupet reconstruction. *Chi-Squared or Fisher’s exact test. †Mann–Whitney *U* test. PPI, proton pump inhibitor; POH, para-oesophageal hernia. Numbers are *n* unless stated otherwise.

### Peri- and postoperative courses

Most operations were performed with a laparoscopic approach, with a conversion rate of 6 per cent (*n* = 2) and 3 per cent (*n* = 1) in the Nissen and the Toupet groups respectively. Only three patients (9 per cent) in the Nissen group and six (15 per cent) in the Toupet groups underwent open procedures (*Table 2*). A Crura-Soft^®^ large mesh was applied in one (3 per cent) patient in the Nissen group and two (5 per cent) patients in the Toupet group to reinforce the hiatal closure. All operations were completed without any major intraoperative complications. The median operative time for a Nissen procedure was significantly shorter than for a posterior partial fundoplication (*Table 2*).

**Table 2:** Peri- and postoperative courses after surgery in patients operated for para-oesophageal hernia

		Nissen	Toupet	*P*
		*n* = 32	*n* = 38	
**Abdominal access**	**Laparoscopy**	27 (85%)	31 (82%)	0.576*
	**Open**	3 (9%)	6 (15%)	
	**Converted to open**	2 (6%)	1 (3%)	
**Duration of operation Minutes, median (i.q.r.)**		126 (101–165)	165 (120–205)	0.021†
**Mesh**		1 (3%)	2 (5%)	0.659*
**Postoperative complications graded according to Clavien–Dindo**	**I**	3 (9%)	1 (3%)	0.454*
	**II**	1 (3%)	3 (8%)	
	**IIIa**	1 (3%)	2 (5%)	
	**IIIb**	3 (9%)	8 (21%)	
	**IVa**	1 (3%)	2 (5%)	
**Clavien–Dindo > II**		5 (15%)	12 (31%)	0.121*
**Acute re-operation**		2 (6%)	3 (8%)	0.790*
**Duration of hospital stay (days), median (i.q.r.)**		2 (2–5)	4 (2–8)	0.129†

Patients were randomized to either a Nissen or Toupet reconstruction. Values are *n* (%) unless stated otherwise. *Chi-Squared or Fisher’s exact test. †Mann–Whitney *U* test.

No difference in overall postoperative complications was observed between the two groups (*Table 2*), even when only morbidity with a Clavien–Dindo score higher than II was considered. No difference was observed between groups in re-operation rates. One patient in the Nissen group and two in the Toupet group were re-operated on due to early re-herniation during the first 2 weeks after surgery. Of two patients who required emergency re-operations, one was in the Nissen group (gastric ischaemic lesion on postoperative day 12), and one was in the Toupet group (oesophageal perforation on a postoperative day 3). In the first postoperative week, one patient in the Nissen group and five in the Toupet group underwent gastroscopy under general anaesthesia for early postoperative dysphagia, and four of these patients underwent endoscopic dilatation (one in the Nissen and three in the Toupet group, *P* = 0.135). Due to respiratory insufficiency, one patient in the Nissen group and two patients in the Toupet group required a short stay in the ICU (*P* = 0.660). The median (i.q.r.) postoperative duration of hospital stay was 2 (2–5) and 4 (2–8) days in the Nissen and Toupet groups respectively (*P* = 0.129). There was no 90-day postoperative mortality.

### Dysphagia

#### Ogilvie score

Ogilvie scores were similar in the two groups at baseline (*Table 3*). At the first postoperative assessment (1 month), no significant improvement was seen in Ogilvie scores in any group. At the 3- and 6-month follow-up, however, the swallowing difficulties improved significantly in the Toupet group (from baseline mean (s.d.): 1.4  (1.1) to 0.5 (0.8) at 3 months, and 0.5 (0.6) at 6 months; *P* = 0.003 and 0.001 respectively). There was no significant difference in Ogilvie dysphagia scores between the two groups at any time point of the follow-up. When the absolute differences in Ogilvie dysphagia scores (⍙) from baseline to the different postoperative time points (1, 3, and 6 months) were considered, a significant difference was observed between the two groups at 6 months (mean (s.d.): ⍙: −0.4  (1.0) in the Nissen group *versus* −1.0  (1.1) in the Toupet group; *P* = 0.032) (*Table S1*).

**Table 3:** Ogilvie dysphagia score before and at 1, 3, and 6 months after surgery in patients operated for para-oesophageal hernia

Allocation	Before surgery (baseline)		1 month after surgery		3 months after surgery		6 months after surgery	
	Nissen	Toupet	Nissen	Toupet	Nissen	Toupet	Nissen	Toupet
	(*n* = 23)	(*n* = 25)	(*n* = 28)	(*n* = 29)	(*n* = 25)	(*n* = 28)	(*n* = 26)	(*n* = 27)
**Ogilvie dysphagia score**	1.1 (1.1)	1.4 (1.1)	1.2 ( 0.8)	0.8 (0.9)	0.6 (0.6)	0.5 ( 0.8)	0.7 ( 0.8)	0.5 (0.6)
*P* between groups*	0.263		0.107		0.474		0.947	
***P versus* baseline**†			0.857	0.101	0.075	0.003	0.084	0.001

Patients were randomized to either a Nissen or Toupet reconstruction. Dysphagia was scored (0–4), where 0 corresponds to no swallowing difficulties and 4 represents complete obstruction. Mean (s.d.) are given. *Mann–Whitney *U* test. †Friedman’s and Wilcoxon signed rank test.

#### Dakkak score

**Fig. 2:**
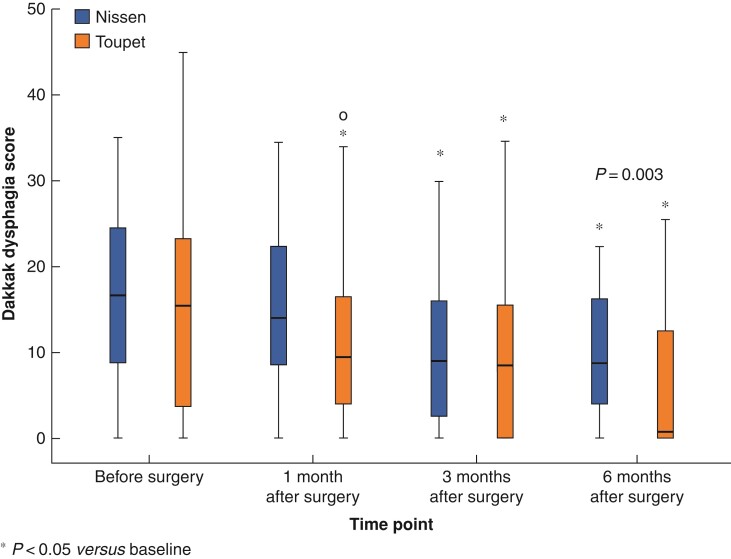
Dakkak dysphagia score before and at 1, 3, and 6 months after surgery in patients operated for para-oesophageal hernia

Dakkak dysphagia scores are presented in *Fig. 2*, and both groups displayed a gradual improvement in scores during the first 6 monthsafter surgery. Compared with baseline, overall scores were significantly improved in the Toupet group by 1 month and in both groups at 3 and 6 months. The Toupet group also demonstrated a significantly lower Dakkak dysphagia score at 6 months than that in the Nissen group (mean (s.d.): 5.1  (7.2) *versus* 10.4 (7.9); *P* = 0.003).

Patients were randomized to either a Nissen or Toupet reconstruction. Box-whisker plots indicate the median value (horizontal line), 25th and 75th quartiles (box), and 10–90-percentile range (whiskers). A circle depicts outliers. *P < 0.05 *versus* baseline.

### Quality of life, acid reflux variables, and PPI consumption

The QoL assessed by SF-36 is reported in *Table 4*. A significant improvement in the PCS was observed in the Nissen group at 3 and 6 months (*P* = 0.021 and *P* = 0.003 respectively). Conversely, PCS, and MCS were both significantly improved in the Toupet group at the same time points. There were no differences in PCS or MCS scores between the two groups at any time point. The absolute differences (⍙) between pre-and postoperative SF-36 values at 1, 3, and 6 months are reported in *Table S2*.

**Table 4:** SF-36 outcomes (physical and mental component score) before and at 1, 3, and 6 months after surgery in patients operated for para-oesophageal hernia

	Nissen	*P versus* baseline Nissen†	Toupet	*P versus* baseline Toupet†	*P* between groups*
**Preoperative (baseline)**	*n* = 31		*n* = 34		
PCS	36.8 (30.7–47)		35.6 (29.3–48.8)		0.793
MCS	50.8 (39.3–53)		40.8 (32.3–50.9)		0.085
**1 month postop.**	*n* = 27		*n* = 30		
PCS	38.4 (31.6–46.2)	0.790	41.1 (34.4–48.2)	0.428	0.565
MCS	43.7 (35.9–52.8)	0.112	44.3 (31.1–50.1)	0.792	0.737
**3 months postop.**	*n* = 26		*n* = 28		
PCS	46.8 (34.9–52.7)	0.021	45.1 (37.1–54.7)	0.026	0.678
MCS	52.7 (37–54)	0.798	52.9 (37.7–57.5)	0.005	0.226
**6 months postop.**	*n* = 28		*n* = 31		
PCS	45.5 (38.2–52.6)	0.003	49.6 (38.3–55.8)	0.000	0.255
MCS	47.4 (36.8–53.6)	0.866	54.4 (39.9–57.8)	0.002	0.066

Patients were randomized to either a Nissen or Toupet reconstruction. Scores are median (i.q.r.). *Mann–Whitney *U* test. †Friedman’s and Wilcoxon signed rank test. PCS, physical component score; MCS, mental component score.

The Toupet group showed significant improved MCS scores at 3 and 6 months after surgery, compared with the Nissen group (median (i.q.r.): 7.1 (−0.6–15.2) *versus* 1.0 (−5.4–3.3) at 3 months, *P* = 0.010; and 11.2 (1.4–18.3) *versus* 0.4 (−9.4–7.5) at 6 months; *P* = 0.003).

**Fig. 3:**
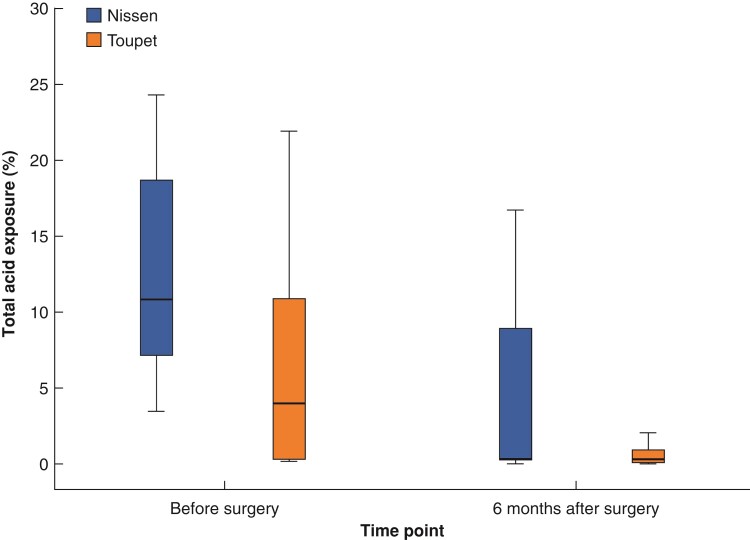
Oesophageal 24-h total acid exposure before and 6 months after either a Nissen (*n* = 10) or a Toupet (*n* = 20) fundoplication during hiatal repair in patients with para-oesophageal hernia

Patients of both groups undergoing pre-and postoperative ambulatory 24-h pH monitoring (10 in the Nissen and 20 in the Toupet group) demonstrated decreasing trends in total oesophageal acid exposure time from baseline (median (i.q.r.): 10.9 per cent (4.3–16.3 per cent) in the Nissen and 8.0 per cent (0.2–11.4 per cent) in the Toupet group) to 6 months postoperatively (median (i.q.r.): 0.25 per cent (0.1–8.2 per cent) in the Nissen and 0.3 per cent (0–1.9 per cent) in the Toupet group). No difference was observed between the two groups (*P* = 0.947) (*Fig. 3*).

Box-whisker plots indicate the median value (horizontal line), 25th and 75th quartiles (box), and 10–90-percentile range (whiskers).

Before surgery, 17 (61 per cent) and 22 (71 per cent) patients were treated with chronic PPI therapy in the Nissen and Toupet groups respectively. The number of patients in the Nissen group requiring PPIs to control GORD symptoms at 1, 3, and 6 months after surgery was 9 of 28 (32 per cent), 9 of 26 (35 per cent), and 8 of 27 (30 per cent) patients respectively. In the Toupet group, there were 10 of 30 (33 per cent), 8 of 30 (27 per cent), and 6 of 29 (21 per cent) patients respectively, without significant differences between the two groups at any of these time points (*P* = 0.672, *P* = 0.489, and *P* = 0.589 respectively).

A subgroup analysis to see whether patients without preoperative GORD were at higher risk of suffering from dysphagia after POH repair was undertaken by comparing postoperative changes in Ogilvie scores (assessed at 6 months) in patients with and without preoperative PPI therapy in each group. No difference was apparent (*P* = 0.141 in the Nissen, and *P* = 0.133 in the Toupet group).

### Hiatal hernia recurrence

At 6 months, 11 of 24 patients (46 per cent) in the Nissen group and 15 of 32 (47 per cent) patients in the Toupet group had a radiologically confirmed hernia recurrence (*P* = 0.938). The median (i.q.r.) axial length of the recurred hernias was 3 (3–6) and 4 (3–5) cm in the Nissen and Toupet groups respectively (*P* = 0.540). All recurrences were classified as type I hernias.

An additional subanalysis performed to determine a possible association between obstructive complications and recurrence found no significant difference between the two groups (recurrence *versus* no recurrence) regarding Ogilvie or the Dakkak dysphagia scores at any of the three follow-up time points.

## Discussion

This randomized, double-blind clinical trial involving patients undergoing POH repair showed no significant difference between a Nissen and a Toupet fundoplication in postoperative mechanical side effects, assessed at 6 months through the Ogilvie dysphagia score. At the same time point, however, the Toupet group demonstrated a significantly lower Dakkak dysphagia score (considered to be a more comprehensive and refined assessment of swallowing functions) than the Nissen group. Similarly, other secondary endpoints showed improved swallowing functions, favouring a Toupet fundoplication after a laparoscopic POH repair.

Side effects after fundoplication among patients with GORD include dysphagia, inability to belch, or vomit, postprandial fullness, bloating, pain, and socially embarrassing flatus^8,10^. Previous studies have demonstrated that a Nissen fundoplication is more frequently followed by mechanical side effects than a partial anterior or posterior (Toupet) technique^31–33^. Possible mechanisms underlying these differences include specific effects linked to the different procedures on physiological mechanisms of reflux prevention such as LOS tone, LOS ability to relax in response to appropriate stimuli, and transient LOS relaxation frequency^34^.

It has been suggested that Nissen fundoplication might overcorrect the mechanical deficiencies of the GOJ in patients with GORD generating a supracompetent gastric cardia^35^. As highlighted in this trial, these mechanical consequences could explain the differences in obstructive symptoms between the two types of fundoplication. In addition, early postoperative mechanical alterations following fundoplication procedures in POH repair change during the first year after the operation^36^ and in the present study, although swallowing function, evaluated by the Ogilvie score, remained unchanged at 1 month after the surgical procedure in both groups, it improved significantly after 3 and 6 months in patients with a Toupet fundoplication. The present results are consistent with those reported in previous studies on patients with GORD, which reported lower mechanical side effects after a Toupet fundoplication, even with long follow-up periods^32,33^.

GORD is a frequent symptom in patients with POH, as seen by the number of patients on chronic PPI treatment in the present trial (56 per cent). Most reports suggest that up to 30 per cent of patients with POH do not display a previous history of GORD^7,11–13^. Routine performance of a total fundoplication on a repositioned but normally functioning GOJ could induce severe obstructive complaints in those patients with POH ^10,35^.

A subanalysis in the present study, based on preoperative PPI therapy as a GORD indicator, to examine the relevance of pre-existing GORD to the functional postoperative outcome found no increase in postoperative obstructive symptoms related to previous PPI use compared with patients not using PPIs. These data should be interpreted with caution, because of the small number of enrolled patients and the uncertainty around using PPIs as an indicator of GORD^37–39^.

Several studies have reported improved QoL after antireflux surgery and POH repair^40–43^. In the present trial, the absolute differences (⍙) between preoperative MCSs of SF-36 and their values at 3, and 6 months after surgery supported the Toupet technique. These results may underscore the clinical relevance of functional and procedure-related differences in patients with POH. This is especially true as previous studies have been unable to demonstrate any differences in QoL when comparing different types of fundoplications in patients suffering from GORD, despite definite imbalances in obstructive complications^16–18,32,44^.

Although considered a crucial clinical parameter after surgical POH repair, hernia recurrence remains a challenge^45,46^. Previous studies have reported radiologically confirmed recurrence rates as high as 50 per cent^47,48^, yet despite such high recurrence rates, the same studies demonstrated significant improvement in QoL, symptom relief, and reflux control^41,47,48^. The pathogenesis of POH recurrence and the factors influencing the hiatal reconstruction durability are still unclear. Despite the short follow-up interval in the present trial, anatomical recurrence rates were high, regardless of the type of fundoplication added to the crural repair. It should be emphasized, however, that in both groups, most hernias were smaller in size at 6 months, and were mostly type 1 sliding hernias rather than POH recurrences. No correlation between obstructive complications and recurrence was observed.

The major strength of this trial is the double-blind, randomized design carried out throughout the entire study interval. Limitations include the incomplete data sets of both pre-and postoperative follow-up parameters. Despite the advantages of elective surgery, mainly linked to the lower complication burden, emergency operations are unavoidable, and in this series about 20 per cent of the patients belonged to this latter category, leading to difficulties in baseline disease-specific assessments. The high proportion of elderly patients with multiple co-morbidities also hampered follow-up data collection. As the primary outcome was swallowing difficulties, as scored by the Ogilvie scale, the problem of incomplete postoperative data was managed by reviewing patients’ medical records. It is reasonable to expect that major swallowing difficulties (persistent or recurrent after the surgical procedure) should have led to further medical consultations, but none of the patients with missing follow-up data attended for medical consultations due to difficulties in swallowing, suggesting that their results are valid and representative for all included patients.

No oesophageal bougies were used in this trial as it is not standard practice in our department. It may be argued that this practice can explain the differences in postoperative dysphagia, but its routine use is now considered optional during antireflux surgery^49–51^.

The long enrolment interval highlighting the low number of procedures performed per year in each included centre adds a further limitation. Although all surgeons involved had significant experience in hiatal surgery, the volume–outcome quality relationship has to be addressed. During recent years the management of patients with POH has been progressively centralized, potentially increasing the quality of surgical care^52^.

## Supplementary Material

zrac034_Supplementary_Data

## Data Availability

The data that support the findings of this study are not publicly available as we have no permission from the ethical review board to share data containing information that could compromise privacy and consent of research participants. However, data are available from the corresponding author upon reasonable request and with permission of the ethical review board. The corresponding author had full access to all data in the study and takes responsibility for its integrity and the accuracy of data analysis.
