# Supplementary material for: Total versus partial posterior fundoplication in the surgical repair of para-oesophageal hernias: randomized clinical trial
Source: BJS Open. 2022 May 2;6(3):zrac034. doi: 10.1093/bjsopen/zrac034 (PMC9070466; doi:10.1093/bjsopen/zrac034)
Supplement: zrac034_Supplementary_Data [file zrac034_supplementary_data.zip › Supplementary_material.docx]

# Supplementary Material: The original study protocol and an English-language version.

# Jämförande studie mellan total och posterior partiell fundoplikatio vid rekonstruktion pga paraesofageal herniering.

**Bakgrund:**

Paraesofageal herniering ay hela eller delar av ventrikeln in i thoraxkaviteten är ett tillstånd som inte sällan ger diffusa och svårtolkade symtom men som kan ge direkt livshotande komplikationer i form av inklämning och cirkulationsstörning. Dessa tillstånd karakteriseras av att en stor defekt föreligger i hiatus med kraftig separation av bada crus fran varandra. Till detta kommer att patienterna manga gånger tvingas att opereras akut eller ”halvakut” och att det då kan vara svart att i detalj utreda bakomliggande patofysiologiska faktorer liksom graden och omfattningen av symtomen.

Traditionellt finns uppgifter i litteraturen om att cirka 50 % av dessa patienter sedan tidigare lider av olika svårighetsgrader av kronisk gastroesofageal reflux. Detta förhållande har påverkat den kirurgiska strategin vid rekonstruktionen av hiatus. Huvudsakligen syftar operationen att återställa anatomin i hiatus, ta bort bråcksäcken samt försnäva och förstärka hiatus sa att en re-herniering förhindras. Traditionellt har detta gjort med s.k. crura plastiker dar icke resorberbart sutur material använts men under senare år har olika syntetiska eller resorberbara nät använts. Utöver detta ar traditionen att en s.k. fundoplikation bor göras dels för att förstärka hiatusplastiken, dels att behandla den bakomliggande refluxsjukdomen (se ovan). Den internationellt mest brukade fundoplikations typen ar en s.k. Nissen fundoplikation, då 360 av distala esofagus omkrets omsluts av fundoplikationen. Fördelen med denna metod

är att den ger en god och långvarig kontroll av refluxsjukdomen. Nackdelen är att en motsvarande operation, utförd på den hälft av patienterna som ej har reflux, kan ha mycket ogynnsamma effekter i form av dysfagi och andra funktionsstörningar eller biverkningar (gas spänningar, svårigheter att rapa osv). En alternativ kirurgisk strategi är att göra enbart en bakre partiell (180') fundoplikatur som ger mindre mekaniska biverkningar och då hypotetiskt skulle vara mer gynnsamt i den grupp av dessa patienter som från borjan inte har refluxsjukomen.

# Hypotes:

Bakre partiell fundoplikation (180‘) utförd i samband med rekonstruktion för akut eller halvakut paraesofageal herniering ger färre mekaniska-funktionella biverkningar än en total fundoplikation enligt Nissen.

# Utförande:

Patienter som opereras akut eller ”halvakut” pga paraesofageal herniering erbjuds att delta i studien. Efter informerat samtycke inhämtas uppgifter om symtom (se protokoll), en CT buk och thorax görs samt om möjligt en kontrast-rtg av esofagus och ventrikeln for att bättre karakterisera hemieringen om man ex. misstänker en betydelsefull glidbråckskomponent.

Därefter randomiseras patienten (i block om 10 patienter, vardera). Operationen kan utforas antingen med öppen eller med laparoskopisk teknik. Efter att bråcksäcken exciderats, hiatus och proximala ventrikelanatomin dissekerats görs en främre resp bakre crusaplastik med icke resorberbart stuturmaterial. Gastrica brevis karlen delas for att trygga en saker och spänningsfri fundoplikatur. Baksidan av den partiella fundoplikationen fixeras med 2-3 suturer till vardera sidans crus. Om det inte bedömes nödvändigt ska ej gastrotomi anlaggas. Om man av nâgon anledning bedömer att ett förstärkande — täckande nät-mesh maste användas för att undvika spänning i hiatusrekonstruktionen så kan detta tillåtas.

# Utfall:

Huvud variabel: dysfagi grad (enl Olgilvie, 6 månaders postoperativt)

# Sekundär variabler:

- Postfundoplikations besvär
- Ambulatorisk pH-metri
- Basalt LES tonus + nadir tryck under vattensväljning
- Time barium swallow parametrar
- Intakt hiatus rekonstruktion matt vid CT eller kontrastr0ntgen
- Watson dysfagi score
- Livskvalitet enl SF 36

# Powerberäkning:

Med en beräknad dysfagi (grad 1-4) pa 30 % kan detta förväntas reduceras med 50 % om en posterior, partiell fundoplikation istället görs. Detta medför att totalt 50 patienter behöver inkluderas for att en motsvarande skillnad ska kunna påvisas med en 95 % sannolikhet och med en styrka av 80 %.

# Preoperativ utredning

Övre endoskopi med biopsi, ej mer än sex manader gammal om i övrigt acceptabel undersokning, dvs esofagit (ja/nej, LA grad, biopsi), HH axial längd.

pH- och tryckmätning om detta finns tillgängligt liksom manometri. Symtomevaluering

Livskvalitet varderas med SF 36 Informerat samtycke.

# Operation Randomisering

Patienten randomiseras efter kontroll att inga hinder om deltagande i studien föreligger. Randomiserings utfall och operationsmetod hålles blindad postoperativt for såväl patient som personal, liksom dem som följer upp patienten.

Stratifiering om kön, BMI och Barrett förekomst (ja eller nej).

# Operationsteknik

Rutinmässigt skall bägge grupperna behandlas lika i alla avseenden förutom plastiken. Vid laparoskopisk operation betonas följande komponenter:

**Access:** Verres kanyl alternativt öppen teknik. Maximalt insufflationstryck 10-12 mm Hg, efter introduktion av kanyler 10 mmHg.

**Troakarer:** Fenn 10 mm troakarer (fler eller engångs).

**Placering:** En troakar i medellinjen 5-8 cm ovan naveln, två troakarer under respektive höger och vänster revbens båge.

**Dissektion:** Dissektion rutinmässigt med ultracision enbart. Diatermi och clips tillägg endast om situationen kräver.

Vänster leverlob halls undan med retraktor (fler eller engångs).

Stor Babcock tång fattar over EGJ och reponerar hiatusbråcket.

Vagus levergren sparas rutinmässigt men kan delas om detta krävs for plastikens färdigställande

Peritoneum incideras i omentum minus strax ovan vagus levergren, förbi hiatus framkant i

riktning mot fundus.

Crurae friprepareras i valfri ordning ned till confluens.

Periesofageal fridissektion 4-5 cm, retroesofagealt fönster skapas bakom bakre vagusgrenen. Fundus mobiliseras rutinmassigt (totalt/subtotalt) till loshet (floppy), dras bakom esofagus och bakre vagus, skall kunna kvarligga på egen hand utan tendens att åter dra sig tillbaka. Testa plastikens löshet genom lyft av fundusflikarna på respektive sida av esofagus till önskat gradtal, bekraftas av ”flikdoktor”.

**Suturmaterial:** Plastik, silke. Hudsutur, vicryl rapid.

# Partiell fundoplikation

**Mobilisering:** Rutinmassig mobilisering av fundus till erforderlig löshet med delning av breviskarl, antal efter behov, fliken skall kvarligga löst till höger om esofagus, enligt ovan. Fundusflik framdrages (floppy), bekräftas av ”flikdoktor”.

**Suturering:** Tre suturer mellan fundusflik dorsalt och vardera vänster respektive höger crura, med början hogt upp till vänster med 1 till 1,5 cm avstånd. Triangelsutur mellan esofagus baktill, fundusflik craniodorsalt och höger crus/diafragma högst upp.

Bakre cruraplastik om kvarvarande öppning/osuturerad del ned till confluens föreligger efter att plastiken suturerats. Två suturer adaptation av fundusfliken framåt mot esofagus laterala framsida på respektive sida så att plastiken täcker 180-200 gr av circumferensen, de nedre av dessa suturer i nivå med gastroesofageala övergången, främre vagus grenen undvikes.

Plastikens längd 4-5 cm matt framtill efter plastikens färdigställande och noteras. Aven plastikens längd baktill mätes efter att de tre suturerna mot vänster crus applicerats.

# Total fundoplikation

**Mobilisering:** Rutinmässig mobilisering av fundus till erforderlig löshet med delning ay breviskarl, antal efter behov, fliken skall kvarligga löst till höger om esofagus, enligt ovan. Fundusflik framdrages (floppy), bekräftas av flikdoktor.

**Suturering:** Rutinmässig bakre cruraplastik liksom främre sådan om defekten sa framtvingar (se nedan), enkla suturer med 1 till 1,5 cm mellanrum for adaptation av crurae utan stramning. Kvarvarande fritt utrymme i hiatus 30%, esofagus utfyller resterande 70%.

3 (-4) suturer mellan fundusflikarna, varav endast den mest distala också satts i esofagus muskelvägg, främre vagus undvikes. Plastiklängd 1,5- 2 cm matt framtill efter färdigställande. Aven plastikens längd baktill mates efter färdigställande och noteras.

**Hiatus fiirslutning (cruraplastik):** Oavsett om patienten opereras laparoskopiskt eller öppet skall hela bråcksäcken om möjligt exstirperas. Hiatus strukturer liksom den gatsroesofageala övergången dissekeras fri från vidhäftande bråckvavnad samt bada crus liksom ligamentum arcuatum aortae prepareras. Bakre suturer sättes först samt att man strävar efter att placera esofagus i ett så ”normalt” läge som möjligt dvs. att man undviker vinkling av den gastroesofageala Övergången. I allmänhet krävs ett par främre silkesuturer for att fa en god förslutning. Vi rekommenderar frikostighet med att komplettera med en främre frenotomi dels

för att oka exponeringen vid den transhiatala dissektionen av distala esofagus samt bråcksäcken samt att förhindra stramning i cruraplastiken. Om man så bedömer kan pledgets användas för att bättre förankra crurasuturerna samt mesh graft om man har stor erfarenhet av detta.

**Partial Versus Total Fundoplication in the Surgical Repair of Para-esophageal Hernia**

**Background**

Laparoscopic para-esophageal hernia (PEH) repair has been established as a safe and effective treatment for symptomatic patients. Today, most surgeons agree that a fundoplication should be included in the hiatal reconstruction in order to reduce the risk of postoperative gastroesophageal reflux and hernia recurrence. However, what type of wrap that should be recommend is yet to be determined.

One might argue that the overall durability and effectiveness of a partial fundoplication in the control of reflux might be less reliable than a total wrap, but on the contrary, the latter carries the risk of inducing a pseudoachalasia similar situation in PEH patients.

**Hypothesis**

Adding a total wrap on a repositioned esophago-gastric junction (EGJ) would inflict significant obstructive consequences compared to a posterior partial wrap in patients undergoing laparoscopic repair for PEH.

**Study design**

Randomized double blind trial. Patients undergoing acute or elective surgery for symptomatic PEH at Ersta Hospital or Karolinska University Hospital will be eligible for inclusion.

Exclusion criteria: age below 18 years, axial sliding hiatal hernia (type I), previous hiatal hernia surgery, American Society of Anesthesiologists (ASA) score IV or above, esophageal achalasia or specific motility disorder, Zollinger-Ellison syndrome, malignant tumor, inability to give informed consent and unwillingness to participate in the study.

Preoperative investigation: upper gastrointestinal (GI) endoscopy, esophageal high-resolution manometry, ambulatory 24-hour pH monitoring, radiology (upper GI series or CT) and QoL questionnaires (short form -36 = SF-36). For assessment and recording of swallowing difficulties, two validated instruments will be used: the Ogilvie dysphagia score and Watson dysphagia score.

Primary outcome: swallowing difficulties as assessed by the Ogilvie score at 6 months after surgery.

Secondary outcomes: perioperative courses, complications, length of hospital stay, postoperative swallowing difficulties as assessed by the Watson dysphagia score, acid reflux control, QoL and radiologically verified recurrent hiatal hernia (defined as any part of the stomach above the hiatal plane).

Power calculation

In order to detect a 50% difference in the reduction in Ogilvie scores at 6 months between the groups with 95% probability and 80% power in total 50 patients has to be enrolled. In order to compensate for the risk of high dropout rates due to the patient profiles, we aim for 70 patients to be enrolled.

Randomization and blinding

Stratifying according to sex and body mass index (BMI). A computer-generated randomization list in blocks of 10 will be used. The patient, staff and clinical assessors will be blinded to the study group allocation.

I**ntervention**

Nissen fundoplication: A total fundoplication will be constructed in which the right and left part of the wrap will be brought together in front of, and slightly to the right of the esophagus, and sutured with three interrupted stitches of 2-0 unabsorbable sutures from the GEJ and cranially to attain a length between the top and bottom sutures of at the most 2 cm. At least one wrap suture has to include the esophageal muscle-wall.

Toupet fundoplication: The wrap will be pulled dorsally around the distal part of the esophagus and GEJ, which will be encircled approximately 180-200 degrees. First, the wrap will beanchored with Gore-tex sutures, dorsally to the left crus with 3 sutures and then to the right crus with another 3 sutures.

Finally, the wrap will be completed with 3-4 sutures, between the edges of the wrap and the right and left side of the esophageal wall, respectively.
