# Supplementary material for: Total versus partial posterior fundoplication in the surgical repair of para-oesophageal hernias: randomized clinical trial
Source: BJS Open. 2022 May 2;6(3):zrac034. doi: 10.1093/bjsopen/zrac034 (PMC9070466; doi:10.1093/bjsopen/zrac034)
Supplement: zrac034_Supplementary_Data [file zrac034_supplementary_data.zip › Supplementary_Table_1.docx]

**Table S1**. Absolute differences (⍙) between pre- and postoperative values for Ogilvie dysphagia score at 1, 3 and 6 months after surgery in patients operated for para-oesophageal hernia. Patients were randomized to either a Nissen or Toupet reconstruction. Dysphagia was scored (0-4) according to Ogilvie, where 0 corresponds to no swallowing difficulties and 4 represents complete obstruction. Mean and standard deviation are given.

|  | **1 month postoperatively** | | **3 months postoperatively** | | **6 months postoperatively** | |
| --- | --- | --- | --- | --- | --- | --- |
| **Allocation** | **Nissen**  (n=22) | **Toupet**  (n=24) | **Nissen**  (n=20) | **Toupet**  (n=23) | **Nissen**  (n=20) | **Toupet**  (n=22) |
| **⍙**  **Ogilvie**  **dysphagia score** | 0.0±1.2 | -0.4±1.2 | -0.5±1.1 | -0.8±1.0 | -0.4±1.0 | -1.0±1.1 |
| **p-value between groups^*^** | 0.161 | | 0.249 | | 0.032 | |

* Mann-Whitney U
