# Supplementary material for: Total versus partial posterior fundoplication in the surgical repair of para-oesophageal hernias: randomized clinical trial
Source: BJS Open. 2022 May 2;6(3):zrac034. doi: 10.1093/bjsopen/zrac034 (PMC9070466; doi:10.1093/bjsopen/zrac034)
Supplement: zrac034_Supplementary_Data [file zrac034_supplementary_data.zip › Supplementary_Table_2.docx]

**Table S2.** Absolute differences (⍙) between pre- and postoperative values for SF-36 outcomes (Physical (PCS) and Mental Component Score, MCS) at 1, 3 and 6 months after surgery in patients operated for para-oesophageal hernia. Patients were randomized to either a Nissen or Toupet reconstruction. Scores are given as median (IQR).

|  | **Nissen** | **Toupet** | **p-value between groups^*^** |
| --- | --- | --- | --- |
| **1 month postop.** | n=26 | n=27 |  |
| ⍙ PCS | 1.6 (-6.5–4.9) | 2.9 (-5.9-11.5) | 0.434 |
| ⍙ MCS | -3.2 (-8.0-2.4) | 1.8 (-11.3-10.6) | 0.213 |
| **3 months postop.** | n=25 | n=26 |  |
| ⍙ PCS | 5.6 (-0.3–10.1) | 2.6 (-1.4-16.3) | 0.880 |
| ⍙ MCS | 1.0 (-5.4-3.3) | 7.1 (-0.6-15.2) | 0.010 |
| **6 months postop.** | n=27 | n=30 |  |
| ⍙ PCS | 5.8 (2.5–11.9) | 7.4 (-0.1-16.6) | 0.263 |
| ⍙ MCS | 0.4 (-9.4-7.5) | 11.2 (1.4-18.3) | 0.003 |

PCS=Physical component score

MCS=Mental component score

* Mann-Whitney U
